# Phylogeography of *Iris loczyi* (Iridaceae) in Qinghai–Tibet Plateau revealed by chloroplast DNA and microsatellite markers

**DOI:** 10.1093/aobpla/plab070

**Published:** 2021-11-01

**Authors:** Guoli Zhang, Yan Han, Huan Wang, Ziyang Wang, Hongxing Xiao, Mingzhou Sun

**Affiliations:** 1 Key Laboratory of Molecular Epigenetics of Ministry of Education, Northeast Normal University, Changchun 130024, China; 2 Qian’an No. 1 Middle School, Tangshan 063000, China

**Keywords:** Genetic diversity, *Iris loczyi*, population structure, Qinghai–Tibet Plateau, refuge

## Abstract

Quaternary climate oscillations and complex topography have tremendous effects on current distribution and genetic structure of species, and hence the Qinghai–Tibet Plateau (QTP), the largest plateau in the world, has become a hotspot for many phylogeographic studies. However, little is known about the phylogeographic pattern of herbaceous plants in QTP. Here, we investigate the genetic diversity, population structure and historical dynamics of *Iris loczyi*, using five chloroplast DNA (cpDNA) fragments and seven microsatellite markers. A total of 15 populations, and 149 individuals were sampled throughout the QTP. High genetic diversity was detected both in cpDNA (*H*_d_ = 0.820) and SSR (*H*_o_ = 0.689, *H*_e_ = 0.699). Ten cpDNA haplotypes and 163 alleles were identified. AMOVA and clustering analyses revealed obvious differentiation between regions. The *N*_st_, *G*_st_ and Mantel test showed significant phylogeographic structure of *I. loczyi*. The neutrality test and mismatch distribution analyses indicated that *I. loczyi* could not have undergone a historical population expansion, but population XS from the Qilian Mountain area could have experienced a local expansion. Bottleneck analyses indicated that *I. loczyi* had not experienced bottleneck recently. Based on cpDNA and SSR results, the Qilian Mountain area was inferred as a potential glacial refuge, and the southern Tibet valley was considered as a ‘microrefugia’ for *I. loczyi*. These findings provided new insights into the location of glacial refuges for the species distributed in QTP, and supplemented more plant species data for the response of QTP species to the Quaternary climate.

## Teaser

As the largest plateau in the world, the Qinghai–Tibet Plateau (QTP) is a hotspot for manyphylogeographic studies, but little is known about herbaceous plants. Here, we revealed the population genetic structure and two possible refuges of *Iris loczyi* distributed in QTP based on chloroplast DNA and microsatellite markers. The geographic isolation caused by multiple mountain ranges was contributed to the current distribution pattern of this species. Our findings provided new insights into the location of glacial refuges for the species distributed in QTP, and supplemented more species data for the response of QTP species to the Quaternary climate.

## Introduction

There is growing evidence that the Quaternary climate oscillations have had tremendous effects on current distribution and genetic structure of species, which may vary with latitude and topography (Hewitt 2000, 2003; Hewitt 2011), with those of the mountainous regions being the most interesting. The role of mountainous regions in nurturing biodiversity is widely recognized (Antonelli 2015; Spicer 2017; Hoorn *et al.* 2018; Rahbek *et al.* 2019a, b), which is fully reflected in the Himalaya, the Hengduan Mountains and now the Qinghai–Tibet Plateau (QTP) (Spicer *et al.* 2020). Among them, the QTP has attracted increasing interest of biogeographers due to its rugged landscape and rich species diversity (Wen *et al.* 2014; Favre *et al.* 2015; Hughes and Atchison 2015).

The QTP is the youngest and largest plateau in the world covering most of Qinghai Province and Tibet of China, with a mean elevation of 4500 m (Zheng 1996), surrounded by several vast mountain ranges (Himalayan, Kunlun, Qilian, Karakoram and Hengduan Mountains) (Rowold *et al.* 2019). A common hypothesis for the rich of mountain biodiversity is uplift-driven diversity, which means that geological events created the conditions for speciation (Hoorn *et al.* 2013; Schwery *et al.* 2014; Favre *et al.* 2015; Hughes 2016; Lagomarsino *et al.* 2016). The uplift of the QTP was one of the most important geological events to have occurred since the Cenozoic (Spicer *et al.* 2003), with the formation of high mountains and deep valleys within the plateau (Li *et al.* 1995) that profoundly influenced the global climate, monsoon intensity and the distribution of species in the region (Raymo and Ruddiman 1992; Zhisheng *et al.* 2001). Therefore, QTP has become a hotspot for many phylogeographic studies (Wen *et al.* 2014), and it may display divergent characteristics in how the Quaternary history (climate oscillations and geological events) affected the current distribution and genetic structure of plants.

Previous studies have revealed two major patterns of plants response to glacial climate change on the QTP: one is that species survived in a refuge on the QTP during the Last Glacial Maximum (LGM), then expanded during the postglacial periods (Wang *et al.* 2009*b*, *c*); the other is surviving in multiple refugia during the LGM throughout the current distribution (Opgenoorth *et al.* 2010; Wang *et al.* 2010). However, they have mainly focused on woody plants or alpine herbs, and little is known about the response of dense perennial herb species growing in sunny environments to glacial climatic oscillations.

*Iris loczyi* (Iridaceae) is a dense perennial herb with knobby rhizomes, linear leaves, tough texture. The flower stem is short, and the base is often surrounded by lanceolate membranous sheath-like leaves. Flowers pale purple, outer segments oblanceolate or narrowly obovate, and inner segments oblanceolate. The capsule is obovate to cylindrical, reddish brown. Flowering from May to June, fruiting from July to September (Zhao 1985; Mathew 1989). This species would be expected to occur only in Xinjiang and western Tibet in China (Flora of China online). However, we found its distribution in eastern Tibet and a large area in Qinghai Province when we collected field samples. *Iris loczyi* is mainly distributed in sunny alpine meadows and desert gravel, and exists in the form of communities, with an altitude of more than 2000 m. *Iris loczyi* has strong adaptability to the harsh environment of the QTP, so it is an important genetic resource for studying the evolutionary history of species on the QTP under climate change and complex terrain conditions.

Here, we conducted a phylogeographic study for *I. loczyi*, employing five chloroplast fragments and seven microsatellite markers. This is also the first phylogeographic study of *Iris* species distributed in QTP by combining chloroplast and nuclear markers. The aims of this study are to (i) estimate the genetic diversity and phylogeographic structure of *I. loczyi*, (ii) infer the potential LGM refugia of *I. loczyi* and determine whether it occupied a single refugium or multiple refugia.

## Materials and Methods

### Plant sampling and DNA extraction

**Figure 1.**
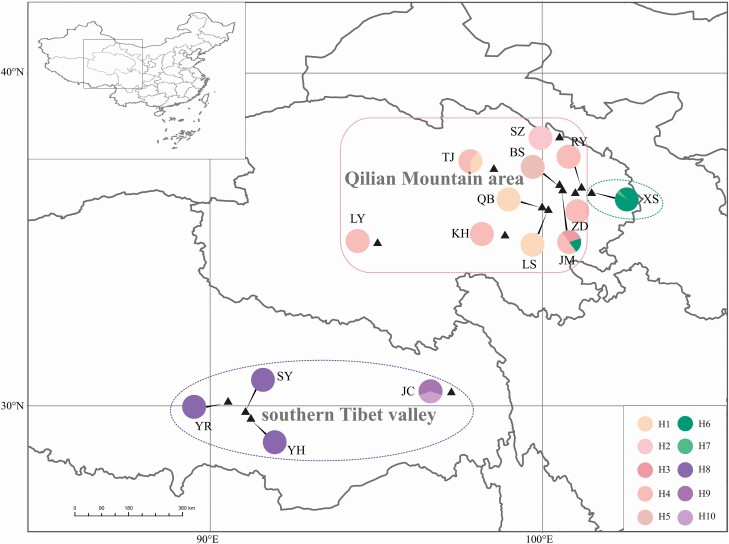
The geographic distributions of sampled *I. loczyi* populations and the cpDNA haplotypes. Section size of the pie reflects the frequency of haplotype occurrence in each population. Each haplotype colour is shown in the bottom right corner legend. The dotted ellipse and rectangle represent the grouping of STRUCTURE analyses (green, Group I; pink, Group II; purple, Group III).

A total of 149 *I. loczyi* leaf samples were collected from 15 populations distributed in eastern Tibet and Qinghai Province, covering most of the geographic range of *I. loczyi* across QTP (Fig. 1). One hundred and thirteen individuals of 11 populations were collected from the Qilian Mountain area, and 36 individuals of four populations from the southern Tibet valley (Table 1). The distance between sampled individuals was ~60 m in the natural populations. Voucher specimens are deposited in the Herbarium of Northeast Normal University (NENU). *Iris goniocarpa* was chosen as an outgroup. Total genomic DNA was extracted using the PlantGen DNA Kits (TianGen, USA).

**Table 1.** Sample information and genetic diversity parameters for 15 populations of *I. loczyi*. QH = Qinghai Province; *S* = number of polymorphic sites; *h* = number of haplotypes; *H*_d_ = haplotype diversity; *π* = nucleotide diversity; *N*_a_ = allele number; *F*_is_ = inbreeding coefficient; *H*_o_ = observed heterozygosity; *H*_e_ = expected heterozygosity. The codes of Group I−III represent the grouping of STRUCTURE analyses.

Code	Location	Size	cpDNA						nSSR			
			S	h	*H*_d_	*π*	Haplotypes		*N*_a_	*F*_is_	*H*_o_	*H*_e_
Qilian Mountain area												
Group I		18	1	2	0.111	0.00004	H6, H7		7.143	0.228	0.556	0.693
XS	Xishan Bay, QH	18	1	2	0.111	0.00004	H6, H7		7.143	0.228	0.556	0.693
Group II		95	86	6	0.677	0.00195	H1–H6		18.429	0.098	0.743	0.828
BS	Baishugou, QH	9	0	1	0.000	0.00000	H5		5.857	−0.044	0.762	0.732
JM	Jiangmu Mountain, QH	10	79	3	0.688	0.00934	H3, H4, H6		7.429	0.067	0.743	0.808
KH	Bitter Sea, QH	10	0	1	0.000	0.00000	H4		6.714	0.120	0.657	0.742
LY	Laiyang Village, QH	10	0	1	0.000	0.00000	H4		6.000	−0.105	0.829	0.754
LS	Lanshuilong, QH	10	0	1	0.000	0.00000	H1		6.286	0.056	0.714	0.754
QB	Qingbao River, QH	10	0	1	0.000	0.00000	H1		6.286	0.176	0.614	0.738
RY	Riyue Township, QH	8	0	1	0.000	0.00000	H4		6.714	−0.155	0.893	0.781
SZ	Qingshizui Town, QH	10	0	1	0.000	0.00000	H2		6.143	−0.055	0.771	0.738
TJ	Tianjun County, QH	10	1	2	0.533	0.00018	H1, H4		6.571	−0.081	0.786	0.730
ZD	Gonghe County, QH	8	0	1	0.000	0.00000	H4		6.571	0.081	0.685	0.741
Southern Tibet valley												
Group III									8.143	0.090	0.583	0.639
JC	Jizhong Township, Tibet	10	1	2	0.533	0.00018	H9, H10		3.857	−0.075	0.557	0.520
SY	Shayinxema, Lhasa, Tibet	8	0	1	0.000	0.00000	H8		3.714	0.039	0.571	0.582
YH	Lhasa Yanghu Lake, Tibet	8	0	1	0.000	0.00000	H8		4.000	0.025	0.554	0.567
YR	Yangre Village, Tibet	10	0	1	0.000	0.00000	H8		4.857	−0.073	0.643	0.599
Total		149	108	10	0.820	0.00981	H1–H10	Mean	5.873	0.014	0.689	0.699

### Chloroplast DNA amplification and sequencing

We amplified and sequenced five chloroplast fragments selected from 56 primer pairs collected from previous research **[see****Supporting Information—Table S1****]**, namely *trnS-trnG*, *trnS-trnfM*, *rpl20*-5*′rps12*, *psaI-accD* and *trnH-pbsA* (Demesure *et al.* 1995; Hamilton 1999; Shaw *et al.* 2007). The PCR amplification was performed in a 30-μL reaction volume containing 20–50 ng template DNA, 10× PCR buffer (Mg^2+^), 10 mM dNTP mixture, 0.1 μM forward and reverse primers and 2.5 U r*Taq* DNA polymerase (TaKaRa, Dalian, China). PCR amplification was performed under the following conditions: 5 min predenaturation at 95 °C; followed by 35 cycles of 30 s denaturation at 94 °C, 30 s annealing at 48 °C (47 °C for *trnH-pbsA*), 60 s elongation at 72 °C and final elongation step of 10 min at 72 °C. All PCR products were detected by electrophoresis on 1.5 % agarose gels and sequenced with the ABI3730 sequencer (Shenggong Biological Engineering Co., Ltd, Shanghai, China).

### Microsatellite genotyping

A total of seven microsatellite markers were used in this study **[see****Supporting Information—Table S2****]**, three (IEST5, IEST8 and IEST12) of which were selected from previously published SSR markers in *Iris laevigata* (Sun *et al.* 2012) and were detected to be polymorphic in *I. loczyi*, with the remaining four (IK2, IK6, IK12 and IK13) were selected from primers independently developed by our team. PCR were performed in volume of 20 μL containing 20–50 ng DNA, 10× buffer (Mg^2+^), 10 mM dNTP mixture, Bovine Serum Albumin, 0.1 μM forward and reverse primer and 2.5 U r*Taq* DNA polymerase. PCR amplification was performed under the same conditions as chloroplast DNA (cpDNA), except for the annealing temperature **[see****Supporting Information—Table S2****]**. The amplified fragments were separated on an ABI 3730 DNA sequence (Applied Biosystems) capillary electrophoresis instrument, and the sizes were assessed using GENEMAPPER 3.7 (Applied Biosystems). To reduce score error, allele size determination was performed twice.

### Genetic diversity and structural analyses

For cpDNA data, the sequences of each primer pairs alignment were performed in MAFFT 7.222 (Kazutaka and Daron 2013) and edited manually in BioEdit 7.0.1 (Hall 1999). The concatenate of sequences were performed by Perl script. Genetic diversity parameters, including the number of polymorphic sites (*S*), number of haplotypes (*h*), haplotype diversity (*H*_d_) and nucleotide diversity (*π*), were calculated using DnaSP 5.10 (Rozas *et al.* 2003). The program PermutCpSSR 2.0 (Hartnup *et al.* 2011) was used for evaluating differentiation for ordered alleles (*N*_ST_) and unordered alleles (*G*_ST_). To determine the best grouping for all populations, Spatial Analysis of Molecular Variance (SAMOVA) analyses (Dupanloup *et al.* 2002) were performed with *K* varying from 1 to 10. Based on the results of SAMOVA, analyses of molecular variance (AMOVA) were performed in Arlequin 3.11 (Excoffier *et al.* 1992), to study the genetic variation partitioned within and among populations/groups. The correlation between genetic (pairwise *F*_ST_ values) and geographical distances was detected using Mantel test in Arlequin, with 1000 randomizations.

The haplotype network was constructed using TCS 1.21 (Clement *et al.* 2000) and the fix connection limit was set as 200 steps with gaps = missing. Bayesian phylogenetic tree of haplotypes was built using MrBayes 3.2.6 (Ronquist *et al.* 2012) under the GTR substitute model. Two Markov chain Monte Carlo (MCMC) searches were run for 500 000 generations each. Trees were sampled every 1000 generations, and the first 25 % was discarded as burn-in.

For SSR data, genetic diversity parameters consisting of mean number of alleles (*N*_a_), inbreeding coefficient (*F*_is_), observed heterozygosity (*H*_o_) and expected heterozygosity (*H*_e_) were calculated using Genepop (http://www.genepop.curtin.edu.au) (Raymond and Rousset 1995) and Popgene (Yeh and Boyle 1996). AMOVA analyses were implemented in Arlequin, and gene flow between population pairs was estimated using Wright’s method, *N*_m_ = (1 − *F*_ST_)/4*F*_ST_ (Wright 1931). STRUCTURE 2.3.3 (Pritchard *et al.* 2000) was used to detect population structure. Six independent simulations were run for each *K* (*K* = 2–8) with 1 × 10^5^ burn-in, followed by 1 × 10^6^ MCMC steps. STRUCTURE HARVESTER (Earl and Vonholdt 2012) was used to determine the optimal *K* value by delta *K* (Δ*K*). Principal coordinate analysis (PCoA) was carried out based on population genetic distance in GenAIEx 6.5 (Peakall and Smouse 2012). The UPGMA phylogenetic tree of populations was constructed using Population 1.2.3 (http://bioinformatics.org/project/?group_id=84) based on Nei’s standard genetic distance between populations.

### Historical population dynamics analyses

To further evaluate the evidence of historical population dynamics, the neutrality test was performed using DnaSP based on cpDNA and the values of Tajima’s *D*, Fu and Li’s *D* and Fu and Li’s *F* (Fu and Li 1993) were calculated. Mismatch distribution analyses (MDA) were performed in Arlequin, with 1000 parametric bootstraps used to test the goodness of fit based on the sum of squared deviations (SSD) and Harpending’s raggedness index (*H*_Rag_) (Rogers and Harpending 1992; Harpending 1994). In addition, the bottleneck was detected using BOTTLENECK 1.2.02 (Cornuet and Luikart 1997) based on SSR data, under the infinite allele model (IAM) and two-phased model of mutation (TPM). One thousand iterations were run. Sign test and Wilcoxon sign-rank test were used to analyse the number of loci with heterozygosity excess and whether the heterozygosity excess was significant.

## Results

### Genetic diversity and population structure based on cpDNA

**Figure 2.**
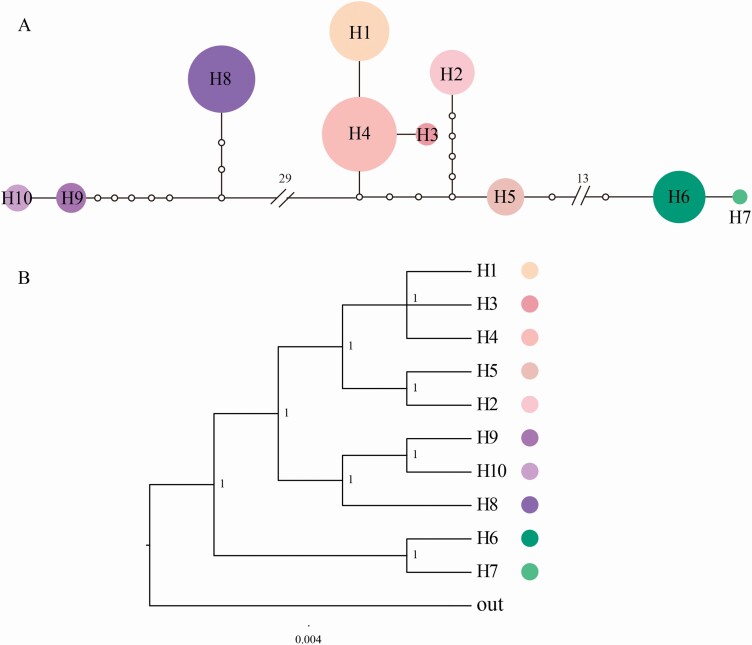
(A) Network of 10 haplotype based on cpDNA of *I. loczyi*. The relative sizes of the circles are proportional to haplotype frequencies. The relative sizes of the circles in the network are proportional to haplotype frequencies. (B) The Bayesian phylogenetic tree of 10 cpDNA haplotypes. Numbers on the nodes represent posterior probabilities values.

The total alignment length of cpDNA sequence was 3445 bp, containing 108 polymorphic sites (Table 1). Ten haplotypes were identified. The total haplotype diversity (*H*_d_) and nucleotide diversity (*π*) were 0.820 (ranging from 0 to 0.688) and 0.00981 (ranging from 0 to 0.00934), respectively. Population JM from the Qilian Mountain area had the highest genetic diversity (*H*_d_ = 0.688, *π* = 0.00934), and contained the most haplotypes (H3, H4 and H6), followed by populations TJ (*H*_d_ = 0.533, *π* = 0.00018), JC (*H*_d_ = 0.533, *π* = 0.00018) and XS (*H*_d_ = 0.111, *π* = 0.00004), all of which contained two haplotypes (H1 and H4, H9 and H10, H6 and H7, respectively). The remaining 11 populations had only one haplotype. There are different haplotypes distributed in the Qilian Mountain area (H1–H7) and the southern Tibet valley (H8–H10), and no shared haplotypes (Fig. 1). The haplotype network showed that H1–H5 were closely related, H6 was closely related to H7 and H8–H10 were close (Fig. 2A), which was consistent with the result of the haplotype phylogenetic tree (Fig. 2B). All haplotype sequences of *I. loczyi* and the sequences of the outgroup *I. goniocarpa* have been uploaded and deposited in the GenBank database (accession numbers: MZ398313–MZ398362, MZ405099–MZ405102).

SAMOVA analyses indicated that the optimal number of groups was *K* = 4, which means all 15 populations were divided into four groups. Population XS from the Qilian Mountain area clustered separately into a group and the remaining 10 populations (BS, JM, KH, LY, LS, QB, RY, SZ, TJ, ZD) in this region composed a group. Population JC from the southern Tibet valley composed a group and the remaining three populations (SY, YH and YR) were assigned to a group. AMOVA analyses revealed that the majority of genetic variation (92.11 %) existed among populations, while, with grouping based on SAMOVA analyses, the majority of genetic variation (91.14 %) existed among groups. The *F*_ST_ value (0.921) also indicated significant genetic differentiation between populations. PermutCpSSR analysis showed that *N*_st_ (0.856, *P* < 0.05) was significantly higher than *G*_st_ (0.839, *P* < 0.05), which indicated a significant phylogeographic structure. The Mantel test (*r* = 0.49; *P* < 0.01) also showed a significant correlation between genetic distance and geographic distance.

### Genetic diversity and population structure based on SSR

A total of 163 alleles at seven SSR loci were revealed across 149 individuals from 15 *I. loczyi* populations (detailed genotype data were listed in **Supporting Information—Table S3**). Genetic diversity parameters for each locus were estimated **[see****Supporting Information—Table S4****]**. The total number of alleles per locus ranged from 14 (IEST8 locus) to 39 (IK6 locus) with a mean of 23.29. The observed heterozygosity (*H*_o_) ranged from 0.4898 (IK2 locus) to 0.9664 (IK13 locus) with a mean of 0.6817 while expected heterozygosity (*H*_e_) ranged from 0.7135 (IEST12 locus) to 0.9259 (IK6 locus) with a mean of 0.8602. Diversity parameters for each population were listed in Table 1. The number of alleles (*N*_a_) ranged from 3.714 (SY) to 7.429 (JM) with a mean number of 5.873. The *H*_o_ ranged from 0.554 (YH) to 0.893 (RY) with an average value of 0.689, the *H*_e_ ranged from 0.520 (JC) to 0.808 (JM) with an average value of 0.699. The inbreeding coefficient (*F*_is_) ranged from −0.155 (RY) to 0.228 (XS) with an average value of 0.014. Population JM had the highest genetic diversity (*N*_a_ = 7.429, *H*_o_ = 0.743), which was consistent with the result of cpDNA. The genetic diversity of the Qilian Mountain area is higher, compared with the southern Tibet valley.

AMOVA analyses showed that the majority of genetic variation (80.82 %) existed within populations (Table 2). A similar result was observed for grouping by STRUCTURE, where 74.96 % genetic variation resided within populations. Estimates of gene flow between population pairs indicated that gene flow between populations was moderate/frequent (*N*_m_ > 1) in the Qilian Mountain area, and moderate in the southern Tibet valley, but there was low (*N*_m_ < 1) gene flow between populations in the two regions **[see****Supporting Information—Table S5****]**, which were consistent with AMOVA analyses.

**Table 2.** Analyses of molecular variance (AMOVA) of *I. loczyi* populations based on cpDNA and nSSR. VC = variance component; PV = percentage of variation; *F*_CT_ = correlation of haplotypes within groups relative to total; *F*_SC_ = correlation within populations relative to groups; *F*_ST_ = correlation within populations relative to total. *P < 0.01.

	cpDNA			nSSR		
Source of variation	VC	PV	Fixation index	VC	PV	Fixation index
All populations						
Among populations	55.649	92.11	*F*_ST_ = 0.921*	0.584	19.18	*F*_ST_ = 0.192*
Within populations	4.766	7.89		2.460	80.82	
Total	60.416			3.044		
By groupings (*K* = 4 for cpDNA, *K* = 3 for SSR)						
Among groups	88.542	91.14	*F*_CT_ = 0.911*	0.524	15.96	*F*_CT_ = 0.160*
Among populations within groups	3.845	3.96	*F*_SC_ = 0.447*	0.298	9.08	*F*_SC_ = 0.108*
Within populations	4.766	4.91	*F*_ST_ = 0.951*	2.460	74.96	*F*_ST_ = 0.250*
Total	97.153			3.282		

**Figure 3.**
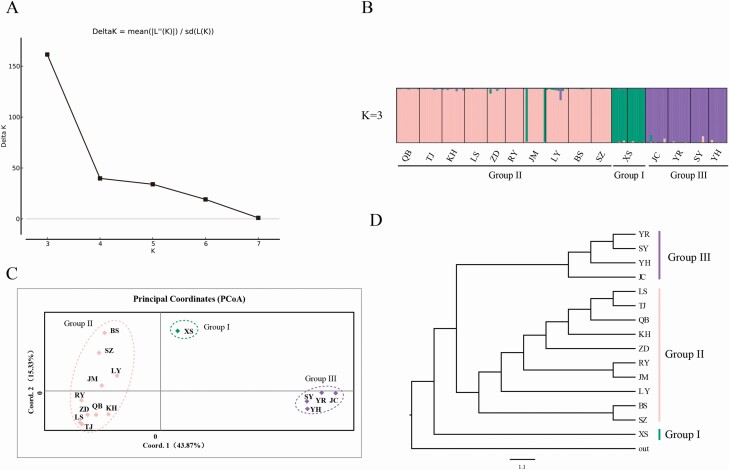
(A) Plot showing delta *K* values for different *K* values tested in STRUCTURE analyses, and *K* = 3 was the optimal value. (B) STRUCTURE clustering results based on *K* = 3. The population and grouping codes were placed at the bottom. (C) Principal coordinate analyses (PCoA) clustering results. The dotted ellipse represents the grouping. (D) The UPGMA phylogenetic tree of 16 populations in *I. loczyi*. The vertical lines on the right represent the grouping.

STRUCTURE analyses revealed that the optimal *K* value was 3 (Fig. 3A), suggesting that the 15 populations were divided into three groups, which was consistent with the results of SAMOVA analyses based on cpDNA, population XS from the Qilian Mountain area was grouped separately (Group I), the remaining 10 populations in this region were grouped (Group II), except that the four populations from the southern Tibet valley were clustered into one group (Group III). In addition, the results suggested the existence of gene flow from population XS to JM (Fig. 3B). The same grouping was found in the PCoA analysis (Fig. 3C). In the UPGMA tree, all populations were grouped into three major clades (Fig. 3D), which were consistent with the results of STRUCTURE and PCoA analyses, and the population relationships showed a correlation with geographic distance.

### Historical population dynamics

**Figure 4.**
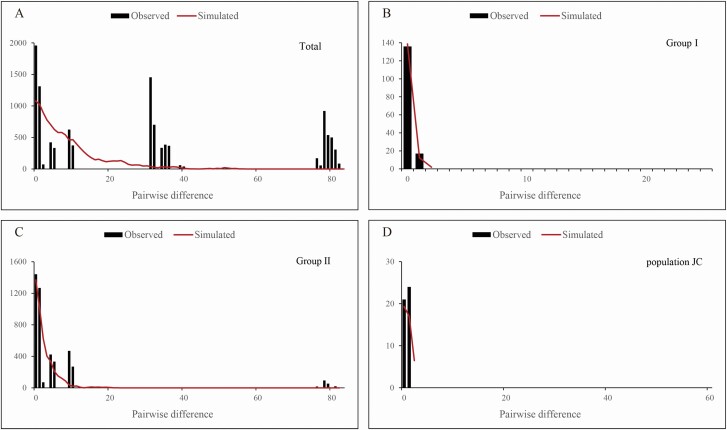
Mismatch distribution of the number of pairwise nucleotide differences for cpDNA data in (A) overall populations, (B) Group I, (C) Group II and (D) population JC. The column and the solid curve represent observed values and simulated values, respectively.

**Figure 5.**
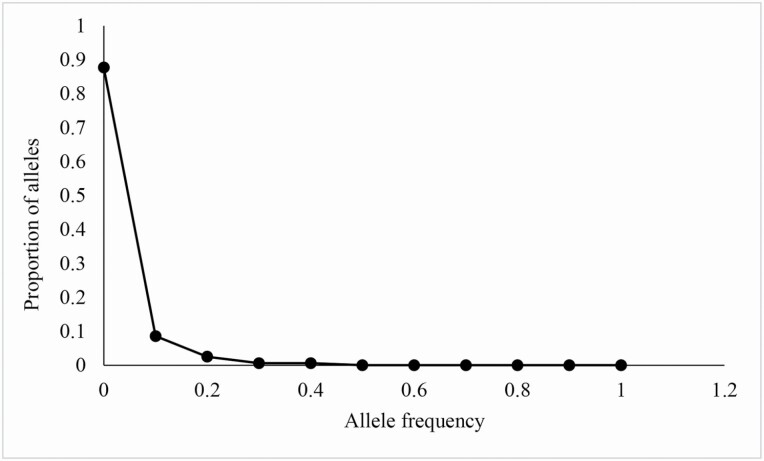
Distribution of allele frequency in BOTTLENECK analyses.

The neutrality test showed that Tajima’s *D* (1.65602, *P* > 0.02) was non-significant positive value while Fu and Li’s *D* (2.61466, *P* < 0.02) and Fu and Li’s *F* (2.61708, *P* < 0.02) were both significant positive values (Table 3), indicating that *I. loczyi* could not have undergone a historical population expansion. Analyses of MDA did not support the expansion hypothesis either, where the mismatch distribution curve showed a multimodal distribution (Fig. 4A). In a separate test of the three groups, Group I presented a non-significant negative value of Tajima’s *D* (−1.16467, *P* > 0.1), and the mismatch distribution curve was unimodal with the non-significant SSD and *H*_Rag_ (*P* > 0.05; Table 3; Fig. 4B), indicating that the population from Group I (XS) could have experienced population expansion. By contrast, Group II and population JC were non-significant negative or positive values of Tajima’s *D* (−2.13501, 1.30268, *P* > 0.02), and MDA analyses showed multimodal (Fig. 4C and D), suggesting that these populations could not have experienced expansion (the group of SY, YH and YR could not be analysed for neutrality test and MDA because these populations contained only one haplotype). Bottleneck analyses showed insignificant heterozygosity excess (*P* > 0.05 both in Sign test and Wilcoxon sign-rank test; **see****Supporting Information—Table S6**), and the allele frequency presented a typical L-shaped distribution (Fig. 5), indicating that *I. loczyi* had not experienced bottleneck recently.

**Table 3.** The results of neutrality test and mismatch distribution analyses. *P < 0.02; **P > 0.05.

Groups	Neutrality tests			Mismatch distribution	
	Tajima’s *D*	Fu and Li’s *D*	Fu and Li’s *F*	SSD (*P*)	*H*_Rag_ (*P*)
Group I	−1.16467	−1.49949	−1.61172	0.00010**	0.61728**
Group II	−2.13501	2.35006*	0.57725	0.04718**	0.10606**
Population JC	1.30268	0.80424	1.02604	0.03032**	0.28889**
Total	1.65602	2.61466*	2.61708*	0.06706**	0.06021**

## Discussion

### Genetic diversity and population structure

In this study, the five cpDNA fragments in *I. loczyi* displayed a high degree of genetic diversity (*H*_d_ = 0.820; Table 1), similar to other herbaceous species distributed in QTP, such as *Stellera chamaejasme* (*H*_d_ = 0.834) (Zhang *et al.* 2010), and species from *Rhodiola* sect. *Trifida* (*H*_d_ = 0.923) (Li *et al.* 2018). Additionally, the degree of genetic diversity was higher than the average level (*H*_d_ = 0.670) of 170 angiosperm species (Petit *et al.* 2005) and other *Iris* species, such as *Iris dichotoma* (*H*_d_ = 0.725) (Zhang *et al.* 2020). SSR data also revealed a high genetic diversity (*H*_o_ = 0.689, *H*_e_ = 0.699), which was generally higher than other studied herbaceous plants in the QTP, e.g. *Rheum tanguticum* (*H*_o_ = 0.342, *H*_e_ = 0.515) (Chen *et al.* 2009), *Elymus sibiricus* (*H*_o_ = 0.269, *H*_e_ = 0.181) (Ma *et al.* 2008) and *Hordeum vulgare* (*H*_o_ = 0.126) (Wang *et al.* 2009*a*), as well as the mean diversity values of perennial plants (*H*_o_ = 0.63, *H*_e_ = 0.68) (Nybom 2004). *Iris loczyi* can either reproduce asexually or sexually, and can inbreeding or outcrossing. The special reproductive system provides the possibility for its high genetic diversity. Population dynamics analyses showed that *I. loczyi* had not experienced population expansion and bottleneck recently, so stable population dynamics is also an important factor for maintaining high genetic diversity. Based on different evidences, some other researchers have suggested that the uplift of mountains was responsible for higher genetic diversity (Xu *et al.* 2010; Qiu *et al.* 2011). Therefore, the orogenic process at QTP can be considered as an essential factor. Theoretically, plant species with a widespread geographical distribution often possess higher genetic diversity, compared with species confined to a restricted distribution (Spicer *et al.* 2003; Yoichi and Tomaru 2014; Levy *et al.* 2016; Chung *et al.* 2018). Compared with the above species, *I. loczyi* has a relatively large geographical distribution in QTP; therefore, this could be another possible factor for the high genetic diversity. The AMOVA analyses of cpDNA showed that the main source of variation originated among populations/groups, while it originated within populations in SSR, the reason was probably due to the fact that the seed flow was limited to the population or the surrounding area by the influence of gravity. And, previous studies have revealed that outcrossing, clonal and long-lived species exhibit relatively high variation within populations (Wróblewska *et al.* 2003).

The differentiation values calculated by AMOVA analyses showed a significant genetic differentiation between *I. loczyi* populations (*F*_ST_ = 0.921 for cpDNA, *F*_ST_ = 0.192 for SSR; Table 2), and the results of the Mantel test also indicated a significant correlation (*r* = 0.49; *P* < 0.01) between genetic and geographic distances. The geographical distribution of haplotypes indicated that there was no shared haplotype between the southern Tibet valley (H8–H10; Fig. 1) and the Qilian Mountain area (H1–H7); furthermore, the haplotype network showed that the haplotypes in the two regions were far from each other (Fig. 2A). Low gene flow (*N*_m_ = 0.814 for SSR) was identified at the level of all populations. Estimates of gene flow between population pairs suggested that there was moderate/frequent gene flow between populations within regions, but low gene flow between the two regions **[see****Supporting Information—Table S5****]**. These findings indicated the existence of geographical isolation between the two regions. Population structure and the level of gene flow can be affected by life history traits and environmental effects (Hamrick *et al.* 1979; Loveless and Hamrick 1984; Nybom 2004; Miao *et al.* 2017). The dispersal of *I. loczyi* pollen is mainly by bees, and the dispersal of seeds is mainly by wind. There are alternating mountain ridges and deep river valleys between the Qilian Mountain area and the southern Tibet valley, which could hinder the transmission of pollens and seeds, resulting in restricted gene flow among populations and increase genetic differentiation between the two regions (Till and Gaudeul 2019). In addition, Quaternary climatic oscillations have been assumed to be the cause of the adaptive population differentiation of some species on the QTP (Ge *et al.* 2005; Xia *et al.* 2005; Yang *et al.* 2008; Wang *et al.* 2009*b*). Thus, the climatic change may also be a potential factor leading to *I. loczyi* differentiation.

Results based on both cpDNA and SSR showed that Group II populations from Qilian Mountain area had the highest diversity (*H*_d_ = 0.677, *N*_a_ = 18.429; Table 1), followed by the southern Tibet valley (*H*_d_ = 0.533, *N*_a_ = 8.143), and Group I had a low diversity (*H*_d_ = 0.111, *N*_a_ = 7.143). The Qilian Mountain area is composed of fault-block mountains and valleys, with numerous peaks, parallel ridges and valleys closely intertwined. The complex topography has contributed to the diversity of populations in the region. For the population XS from Group I, it is located in the Huangshui River Valley Basin, with the Daban Mountain in the north, the Laji Mountain in the south and the Riyue Mountain in the west, surrounded by hills. The geographical barriers limited the dispersal of pollen and seeds, which impeded its communication with other populations; this might have contributed to the low diversity. STRUCTURE analyses indicated that population JM had gene flow from population XS (Fig. 3B), and haplotype analyses based on cpDNA showed that populations JM and XS had shared haplotypes (H6; Fig. 1), suggesting that there might have been a unidirectional seed flow from XS to JM. The MDA analyses showed that XS population could have experienced population expansion (Fig. 4). Thus, the gene flow might have occurred in the process of colonization through dispersal. The XS population is adjacent to the Riyue Mountain in the west, which to a certain extent hinders their communication with other populations in the Qilian Mountain area. Therefore, we can speculate that the gene flow was likely to have occurred in the early stages of the formation of the mountain range.

### Glacial refugia

In the past decade, the response of species on the QTP and its adjacent areas to Quaternary climate oscillations has been extensively studied (Opgenoorth *et al.* 2009; Wang *et al.* 2010; Gao *et al.* 2012; Ren *et al.* 2016). In this study, the neutrality test and MDA analyses both suggested that *I. loczyi* did not experience large-scale population expansion (Table 3; Fig. 4), and only a few populations had experienced local range expansion (Group I). The populations in the Qilian Mountain area and the southern Tibet valley were far apart from each other, and neither of them had experienced expansion events. Therefore, a single glacial refuge can be ruled out, that means, *I. loczyi* might have survived *in situ* in multiple refugia on the QTP during the LGM. Compared with the southern Tibet valley, the Qilian Mountain area has a higher genetic diversity, with H4 having the highest frequency (31.5 %). So, the Qilian Mountain area might have been a refuge of *I. loczyi* during the LGM. On the other hand, the Bayesian phylogenetic tree of haplotypes showed that haplotypes (H8–H10) from the southern Tibet valley were resolved as a sister group to the haplotypes H1–H5 from the Qilian Mountain area. STRUCTURE analyses showed that the populations from the southern Tibet valley were clustered into a separate group. In addition, H8 was an endemic haplotype confine to the southern Tibet valley with high frequency (17.4 %). The distribution of populations with high genetic diversity and frequency of private haplotypes indicated the existence of ‘microrefugia’ throughout the distributional range (Li *et al.* 2018). Thus, the southern Tibet valley might have been a ‘microrefugia’ for *I. loczyi* in LGM.

As the world’s largest and highest plateau, the southern and the south-eastern edges of the QTP, especially the Himalaya and Hengduan Mountains, were considered as the biodiversity hotspots (Myers *et al.* 2000; Mittermeier *et al.* 2004, 2011) and important refugia for most of the plant species (Yang *et al.* 2008; Wang *et al.* 2009*b*; Zhang *et al.* 2012; Khan *et al.* 2014), while the inner and northern parts of the QTP were much less studied. The northern part of QTP is bounded by the Kunlun Mountains and the Qilian Mountains while the characteristic topographic features of the inner region are parallel mountain ridges incised by a deep river valley. The distribution of *I. loczyi* in QTP occupies two different geographical regions, the Qilian Mountain area and the southern Tibet valley, that both were considered as the refugia of *I. loczyi* in the glacial period. These findings provide new insights into the location of glacial refuges for the species distributed in QTP, and the study of historical population dynamics supplements more plant species data for the response of QTP species to the Quaternary climate oscillations.

## Supporting Information

The following additional information is available in the online version of this article—

**Table S1.** Fifty-six pairs of chloroplast universal primer related information. The primers in bold are used in this study.

**Table S2.** Characteristics of seven microsatellite markers for *I. loczyi*.

**Table S3.** Summary of SSR genotype data.

**Table S4.** Genetic diversity information of seven microsatellite loci.

**Table S5.** *N*_m_ values between population pairs of *I. loczyi* based on SSR loci.

**Table S6.** Sign test and Wilcoxon sign-rank test to evaluate *I. loczyi* for mutation drift equilibrium under different models.

plab070_suppl_Supplementary_Tables_S1-S6

## Data Availability

All cpDNA haplotype sequences of *Iris loczyi* and the sequences of the outgroup *I. goniocarpa* used in this study are available from NCBI (https://www.ncbi.nlm.nih.gov/). The GenBank accession numbers are MZ398313–MZ398362, MZ405099–MZ405102. SSR genotype data are available as a supplementary file.

## Notes

**Evolution & Diversity. Chief Editor**: Jeremy Beaulieu
